# Laparoscopic Major Vascular Injuries Report of Two Cases and Review

**DOI:** 10.2147/IMCRJ.S394281

**Published:** 2023-01-05

**Authors:** Nissar Shaikh, Umm e-Amara, Athika Sajeer, Rohma Malik, Shoaib Nawaz, Shajahan Idayathulla, Abdul Gafoor M Tharayil, Abdulqadir J Nashwan

**Affiliations:** 1 Surgical Intensive Care Department, Hamad General Hospital (HGH), Hamad Medical Corporation (HMC), Doha, Qatar; 2 Nursing Department, Hazm Mebaireek General Hospital (HMGH), Hamad Medical Corporation (HMC), Doha, Qatar

**Keywords:** aorta, bowel, bladder, entry injury, hemorrhage, laparoscopy

## Abstract

Laparoscopic surgery is the standard of care for various abdominal pathologies due to its apparent advantages. Unintentional and accidental injuries of the intraabdominal organs and vessels caused by trocars and Veress needles are rare, but potentially fatal complications of laparoscopic surgery. Veress needle and trocar can cause major vascular, bowel, or urinary tract injuries. We report two cases of vascular laparoscopic entry injuries. Laparoscopic entry injuries are rare but life-threatening and can cause multiple organ dysfunctions. Therefore, early recognition, aggressive resuscitation, and appropriate management of laparoscopic entry injuries are vital for better outcomes.

## Introduction

Laparoscopic surgeries have evolved over decades to become a safe and the standard of care for abdominal surgeries. The creation of abdominal pneumoperitoneum is one of the unique initial stages with the Verses needle for laparoscopic surgeries. This Veress needle insertion in the abdomen carries the risk of vascular, bowel, and bladder injuries, and 50% of injuries occur before the surgery’s commencement.^1^ Major vascular injury during laparoscopy is a relatively rare complication. Its incidence has been reported between 0.2% and 0.5% in different studies.^2^,^3^ However, it is one of the most serious complications causing an immediate threat to the patient’s life. Mortality after having a major vascular injury during laparoscopy has been reported between 6% and 13%.^2^ Major vascular injuries are quite rare but during when it occurs, it causes significant mortality and morbidity, they often occur during Veress needle or trocar entry to the abdomen this risk increases especially in slim patients and the most frequently injured vessels are the abdominal aorta, iliac arteries and vena cava. Aydin et al describe a case iliac artery injury during the diagnostic laparoscopy.^3^ The most frequently injured Early detection and interventions with conversion from laparoscopy to laparotomy of these initial laparoscopic injuries are key for better outcomes.^4^,^5^ We report two cases with life-threatening major vessel injuries during routine laparoscopic surgeries. Written informed consent was obtained from both patients for the publication of this case report and any accompanying images. The institutional approval was obtained to publish the case.

## Case 1

### Background

The emergency medical services brought a 35 years old male patient to the hospital emergency department with hemorrhagic shock. The patient had been undergoing a routine laparoscopic appendectomy at a private hospital and was reported to have sustained an iatrogenic vascular injury during the start of the surgery. He did not have any previous medical or surgical history. In addition, there was no history of any bleeding disorders in his family.

The patient was brought to the emergency department in intubated and ventilated condition. He was in hypovolemic shock with a systolic blood pressure of 70mmHG, a pulse of 120 beats/minute, and a saturation of 90% while receiving 100% oxygen. On examination, GCS was 2t. He had cold peripheries and a distended abdomen.

FAST (focus abdominal sonography in trauma) scan was positive for massive fluid in the abdomen. ABG showed severe metabolic acidosis with a pH of 7.0 and lactate of 10. Hemoglobin was 5.0 g/dl, and hematocrit of 19 only. Massive transfusion protocol was activated, and the patient was immediately transferred to the Operation theatre for life-saving laparotomy and control of intra-abdominal bleed.

### Intraoperative Course

An emergency consent was taken for laparotomy under general anesthesia. An arterial line and central venous catheter were inserted. Massive bleeding was discovered from the aorta just above its bifurcation. The abdominal cavity was full of blood. Through and through, injury of the aorta was seen. Piercing injury to the small bowel was seen 80 centimeters proximal to the ileocolic junction. Injury to the mesentery was observed, and a gangrenous appendix was seen. While transfusion was continued during the surgery, the aorta was clamped, and the injury was repaired. Resection of the injured bowel segment was done, and end-to-end anastomosis was made. Appendectomy was completed.

The patient remained on high vasopressor support throughout the surgery. Estimated blood loss was around 5 liters. The patient was in severe metabolic acidosis and hemorrhagic shock. After completing the surgery, the abdomen was left open with a Bogota bag dressing, and the patient was transferred to surgical intensive care unit (SICU) in intubated and ventilated condition for further management.

### Post-Operative Events

The patient was admitted in shock and severe metabolic acidosis with a high lactate and noradrenaline infusion but was still hypotensive and had low urine output. The coagulation profile was deranged with low fibrinogen showing a disseminated intravascular coagulopathy (DIC). Therefore, the 2nd set of massive transfusion protocol blood products was transfused.

Further fluid management was done according to PICCO (Pulse contour Cardiac output) studies. The patient received fibrinogen, tranexamic acid, and sodium bicarbonate infusion to correct the metabolic acidosis. Maximum lactate of 17mmol/L was observed on arterial blood gas analysis with a minimum pH of 6.9. The patient was kept intubated and sedated.

On the 2nd day of the SICU, the patient had ongoing bleeding from the open abdominal wound. He became anuric and metabolic acidosis continued despite management. The patient underwent 2nd surgery, and surgical hemostasis was achieved from the open wound edges.

On the 3rd day, the patient was hemodialyzed because of his kidney failure. He also had two episodes of melena. A 3rd look laparotomy was done in which a spurting artery was ligated near the anastomotic site. A vacuum dressing was applied, and the patient was transferred back to the SICU.

### Patient Outcome

The patient remained on total parenteral nutrition for 7 days. He underwent regular hemodialysis and remained anuric for 8 days in SICU. Vasopressor support was weaned off. The patient was extubated on day 5 in the SICU. He underwent several vacuum assisted continuous (VAC) dressing changes in the operation theatre, and his abdomen was closed with mesh on day 17 in the SICU. A total of 10 sessions of renal replacement therapy (Hemodialysis) were done, after which the patient started producing adequate urine and electrolytes as well, as renal markers came to normal range.

The patient received 28 units of Packed Red blood cells, 26 units of Fresh frozen plasma, and 12 units of platelets in addition to fibrinogen. The patient was transferred to the ward for further care and improvement after staying in the SICU for 20 days.

## Case 2

### Background

A 49 years old female patient was admitted to the hospital for elective laparoscopic oophorectomy for right ovarian dermoid cyst and hysteroscopy for dysfunctional uterine bleeding. She had a background history of heavy smoking and was allergic to penicillin. No other significant medical history was noted.

### Intraoperative Events

Induction of anesthesia was done with propofol, remifentanil infusion, and cisatracurium. After smooth endotracheal intubation, the patient was positioned in a lithotomy position, and a Foley’s catheter was inserted. First, a complete hysteroscopy, including an endometrial biopsy, was performed uneventfully. For oophorectomy, the patient’s position was changed to supine, and a 5 mm incision in the umbilicus was done, followed by Veress’s needle insertion after tenting up the skin by towel clip and confirmed by saline syringe infiltration. After insufflation with carbon dioxide, a trocar was inserted, which showed no injury to internal organs. There was omental and peritoneal adhesion to the anterior abdominal wall. Left lower trocar and cannula insertion were successful.

In preparation for removing the right adnexa, 5 mm trocar from the umbilicus was removed, the incision was increased to 10 mm, and a trocar of the same size was placed. However, blood was immediately seen coming from the major vessel site. Therefore, the surgeon took the decision to do midline incision laparotomy, and the vascular surgeon was called to assist in the surgery.

Meanwhile, fluid resuscitation was started with 2 wide bore intravenous cannulas inserted, and a fast infusion of crystalloids and colloids were started. Severe bleeding was noted from the right common iliac artery. The vascular surgeon tried to achieve hemostasis. Patient had hemorrhagic shock, required blood and blood products. Phenylephrine followed by noradrenaline infusions was started. Sodium bicarbonate was given intravenously to correct a severe metabolic acidosis.

Hemorrhage was controlled, and the right iliac artery was repaired with a graft taken from the great saphenous vein, the ovary showed torsion and needed an oophorectomy. The patient received a total of 18 units of packed red blood cells, 12 units of fresh frozen plasma, 12 units of platelets, and 4 grams of fibrinogen.

### Post-Operative Events

The patient was transferred to the SICU in intubated and sedated, where her condition stabilized relatively quickly. Acidosis improved; hemoglobin and coagulation profile were within normal limits, and all electrolytes were corrected.

### Patient Outcome

The patient was extubated after staying in SICU for 24 hours. She remained vitally stable and was shifted to the ward for further care.

## Discussion

Although laparoscopic surgeries have evolved and have become the standard of care over the years, the rate of major complications, namely vascular, bowel, and urological injuries, have remained rare but constant over the last 3 decades. Vascular injuries are comparatively rare, especially injuries of major vessels like the aorta, which is a retroperitoneal structure.^1^,^4^ In a study from Finland, the overall rate of major trocar injuries was 1.4/1000 procedures, 0.6/1000 intestinal injuries, 0.3/1000 urological injuries, and 0.1/1000 vascular injuries.^6^ Filler et al described that laparoscopic cholecystectomy was associated with a majority of fatal and non-fatal injuries.^4^

Vascular entry injuries are the most feared complications of laparoscopic surgeries and frequently occur with the insertion of a Veress needle or trocar due to the proximity of major abdominal vessels and abdominal wall. The right iliac artery lies just below the umbilicus.^1^ The vascular injuries remained a significant cause of mortality from laparoscopic surgeries.^1^ Bowel injuries are the 3rd major cause of mortality in laparoscopic surgeries, and these injuries go unnoticed and are a reason for the delay in diagnosis and management. The reported mortality from laparoscopic bowel injuries ranges from 2.5% to 5%.^7^

Our first case somehow sustained major aortic injury either with the Veress needle or the trocar insertion, which caused massive blood loss in a short period of time. In such dire emergencies, a threat to life is imminent. Fortunately, the patient was transported from the private hospital with limited access to resources like blood and blood products to a tertiary care center in time; he was resuscitated, and the bleeding was controlled relatively quickly. However, the renal injury was inevitable, given the circumstances of prolonged hypotension and hemorrhagic shock.

In the 2nd case, there was an obvious injury to the right common iliac artery from the 10mm trocar inserted into the peritoneal cavity. Apparently, it was an avoidable injury as the abdomen had already been insufflated with carbon dioxide, and the trocar should have been inserted under vision. Nevertheless, the injury was life-threatening and immediate resuscitation, followed by hemostasis and vascular repair, spared the patient from a worse outcome.

The recommendations to prevent or minimize laparoscopic entry injuries are that surgeon should be adequately trained. He should confirm the intraperitoneal placement of the Veress needle, maintain the intraabdominal pressure of 12–14 mm Hg, avoid excessive manipulation of the needle, and the abdominal cavity should be visualized for any fresh blood or other injuries after the introduction of the needle.^1^

If there is a vascular injury, prompt recognition, assessment of vascular injury, and control of bleeding and vascular repair with the help of vascular surgeons should be done. The resuscitation should be with blood and blood products, preferably with activation of massive transfusion protocols.^2^ The bowel injury should be recognized and repaired early and managed accordingly by the team of acute care surgeons and anesthesiologist is the key to a better outcome.^8^

## Conclusion

The concluding lines from our case report are that laparoscopic entry injuries life-threatening, early recognition and multidisciplinary and organ supportive care is essential for a better patient outcome.

## Data Sharing Statement

All data generated or analyzed during this study are included in this published article.
